# Trends in diabetes medication use in Australia, Canada, England, and Scotland: a repeated cross-sectional analysis in primary care

**DOI:** 10.3399/bjgp20X714089

**Published:** 2021-02-23

**Authors:** Michelle Greiver, Alys Havard, Juliana KF Bowles, Sumeet Kalia, Tao Chen, Babak Aliarzadeh, Rahim Moineddin, Julian Sherlock, William Hinton, Frank Sullivan, Braden O’Neill, Conrad Pow, Aashka Bhatt, Fahurrozi Rahman, Bernardo Meza-Torres, Melisa Litchfield, Simon de Lusignan

**Affiliations:** Department of Family and Community Medicine, North York General Hospital, Toronto, Canada and Department of Family and Community Medicine, Faculty of Medicine, University of Toronto, Toronto, Canada.; Centre for Big Data Research in Health, University of New South Wales Sydney, Sydney, Australia.; School of Computer Science, University of St Andrews, St Andrews, UK.; Department of Family and Community Medicine, Faculty of Medicine, University of Toronto, Toronto, Canada.; Department of Family and Community Medicine, North York General Hospital, Toronto, Canada, and Diabetes Action Canada, Toronto, Canada.; Department of Family and Community Medicine, Faculty of Medicine, University of Toronto, Toronto, Canada.; Department of Family and Community Medicine, Faculty of Medicine, University of Toronto, Toronto, Canada.; Nuffield Department of Primary Care Health Sciences, University of Oxford, Oxford, UK, and Department of Clinical and Experimental Medicine, University of Surrey, Guildford, UK.; Nuffield Department of Primary Care Health Sciences, University of Oxford, Oxford, UK, and Department of Clinical and Experimental Medicine, University of Surrey, Guildford, UK.; Department of Family and Community Medicine, Faculty of Medicine, University of Toronto, Toronto, Canada, and School of Medicine, University of St Andrews, St Andrews, UK.; Department of Family and Community Medicine, North York General Hospital, Toronto, Canada and Department of Family and Community Medicine, Faculty of Medicine, University of Toronto, Toronto, Canada.; Department of Family and Community Medicine, North York General Hospital, Toronto, Canada, and Diabetes Action Canada, Toronto, Canada.; Department of Family and Community Medicine, Faculty of Medicine, University of Toronto, Toronto, Canada.; School of Computer Science, University of St Andrews, St Andrews, UK.; Nuffield Department of Primary Care Health Sciences, University of Oxford, Oxford, UK, and Department of Clinical and Experimental Medicine, University of Surrey, Guildford, UK.; Centre for Big Data Research in Health, University of New South Wales Sydney, Sydney, Australia.; Nuffield Department of Primary Care Health Sciences, University of Oxford, Oxford, UK; director, Royal College of General Practitioners Research and Surveillance Centre, London, UK.

**Keywords:** diabetes mellitus, type 2, drug therapy, electronic health records, pharmacoepidemiology, primary health care

## Abstract

**Background:**

Several new classes of glucose-lowering medications have been introduced in the past two decades. Some, such as sodium-glucose cotransporter 2 inhibitors (SGLT2s), have evidence of improved cardiovascular outcomes, while others, such as dipeptidyl peptidase-4 inhibitors (DPP4s), do not. It is therefore important to identify their uptake in order to find ways to support the use of more effective treatments.

**Aim:**

To analyse the uptake of these new classes among patients with type 2 diabetes.

**Design and setting:**

This was a retrospective repeated cross-sectional analysis in primary care. Rates of medication uptake in Australia, Canada, England, and Scotland were compared.

**Method:**

Primary care Electronic Medical Data on prescriptions (Canada, UK) and dispensing data (Australia) from 2012 to 2017 were used. Individuals aged ≥40 years on at least one glucose-lowering drug class in each year of interest were included, excluding those on insulin only. Proportions of patients in each nation, for each year, on each class of medication, and on combinations of classes were determined.

**Results:**

Data from 238 619 patients were included in 2017. The proportion of patients on sulfonylureas (SUs) decreased in three out of four nations, while metformin decreased in Canada. Use of combinations of metformin and new drug classes increased in all nations, replacing combinations involving SUs. In 2017, more patients were on DPP4s (between 19.1% and 27.6%) than on SGLT2s (between 10.1% and 15.3%).

**Conclusion:**

New drugs are displacing SUs. However, despite evidence of better outcomes, the adoption of SGLT2s lagged behind DPP4s.

## INTRODUCTION

Diabetes is one of the most common chronic conditions worldwide and is associated with elevated risks of morbidity and early mortality.^1^ Medication options for the management of elevated blood sugar have evolved; several new classes of glucose-lowering medications for diabetes have been introduced. Dipeptidyl peptidase-4 inhibitors (DPP4s) have a lower risk of hypoglycaemia than older drugs such as sulfonylureas (SUs).^2^ However, the class does not improve cardiovascular risk compared to placebo.^3^^,^^4^ Sodium-glucose cotransporter 2 inhibitors (SGLT2s) and glucagon-like peptide 1 receptor agonists (GLP1s) have been introduced in the past two decades.^5^^,^^6^ Drugs in both classes have been found to be associated with a reduction in cardiovascular outcomes, decreases in the progression of renal disease, and lower rates of heart failure in high-risk patients with diabetes.^5^^–^^7^ The seminal trials establishing the safety of these agents and their effectiveness at improving cardiovascular outcomes were EMPA-REG for empagliflozin, a SGLT2 (September 2015),^5^ and LEADER for liraglutide, a GLP1 (July 2016),^6^ leading to guideline recommendations to use these classes in people with diabetes who have an elevated cardiovascular risk.^8^^–^^10^ Additional trials have since been published, confirming decreases in cardiovascular events.^11^^–^^13^ In a recent systematic review, SGLT2s and GLP1s were associated with lower mortality compared with DPP4s.^4^

Metformin has traditionally been the first-line medication to reduce glucose and continues to be recommended as such;^8^^–^^10^ some have questioned its effectiveness at reducing all-cause and cardiovascular mortality.^14^ Sulfonylureas are more likely to be associated with hypoglycaemia than other classes of oral medications^8^^,^^9^ and may be associated with an unfavourable risk-to-benefit balance in some older adults (≥65 years).^15^^,^^16^

**Table:** How this fits in

Metformin has been a mainstay of treatment for type 2 diabetes mellitus since 1998, following evidence of better cardiovascular outcomes; this evidence is lacking for sulfonylureas. Some newer drugs, such as sodium-glucose cotransporter 2 inhibitors (SGLT2s) and glucagon-like peptide 1(GLP1), decrease the risk of adverse cardiac and renal outcomes in patients at higher risk, while others, such as dipeptidyl peptidase-4 inhibitors (DPP4s), are not better than placebo. The authors found that older drugs, such as sulfonylureas, are being displaced by newer drugs and that more DPP4s than SGLT2s are used. GPs should consider emerging evidence of outcome benefits when prescribing.

In addition, there is a possible increase in cardiovascular risk when SUs are added to metformin.^17^^,^^18^ Guidelines now recommend combining metformin with SGLT2s or GLP1s as preferred agents when cardiovascular or renal disease is present. These agents should be considered when minimising weight gain or when weight loss is a goal of care.^10^ SGLT2s and GLP1s are associated with weight loss, whereas DPP4s are weight neutral.^10^ The majority of patients with diabetes have a body mass index in the overweight or obese range.^19^^,^^20^ The use of SGLT2s or GLP1s should therefore supersede that of DPP4s owing to indications in more clinical contexts.

Although metformin continues to be a first-line drug, cardiovascular protection using it seems to be less than that observed with SGLT2s or GLP1s.^21^ It is possible that SGLT2s and GLP1s may be preferred as first line in the future,^22^ though this is not yet recommended in guidelines.

Recent observational reports have highlighted significant changes in glucose-lowering medications, including decreasing use of SUs and increasing use of all newer drugs in the US, UK, and Denmark.^23^^–^^26^ The increasing availably of national medication information provides opportunities to follow, compare, and contrast trends in antidiabetic medication use in different nations that share healthcare systems with common features. The differential uptake of new medications should be studied, as there is now evidence of improved outcomes for some treatments (SGLT2s, GLP1s), and not for others (DPP4s).

Australia,^27^ Canada,^28^ and the UK^29^ have high functioning, publicly funded healthcare systems that include primary care as a key element;^30^ most patients with diabetes are followed in primary care in these nations. There is limited information on the uptake of newer medications with differences in outcomes in countries with similar, high-functioning primary care systems, such as those studied in this project.

The objectives of this project were to study trends in diabetes medications in the presence of competing new drugs and changing evidence and guidelines. This was compared in different nations with similar healthcare systems.

## METHOD

A repeated cross-sectional retrospective observational design was used. The strengthening the reporting of observational studies in epidemiology (STROBE) checklist was applied for reporting observational studies.^31^

### Settings and data sources

Data from Australia, Canada, England, and Scotland were obtained and used from 1 January 2012 to 31 December 2017 (from 01 July 2012 to 31 December 2017 for Australia). Analyses were conducted in parallel in each nation. The two UK databases were analysed separately because there have been differences in the health systems of the four nations that make up the UK since 1998.^32^^–^^34^

Canadian and UK databases used routinely collected clinical electronic medical record (EMR) data from primary care databases; these were prescribing data. The Australian database included population-wide pharmaceutical dispensing data.

### Australia

In Australia, all citizens, permanent residents, and eligible foreign visitors, that is, those with reciprocal healthcare agreements with Australia, are entitled to subsidised access to prescribed medications through the Pharmaceutical Benefits Scheme (PBS). In this study, dispensing data for a 10% random sample of PBS-eligible persons were used. This is a standard dataset provided by the Australian Government Services Australia for analytical use.^35^ Since July 2012, PBS data has had complete capture of all dispensing of PBS-listed medications, irrespective of price; only medications priced above the co-payment threshold were recorded before this.

### Canada

The National Diabetes Repository was used, which was created in 2018 using de-identified data from EMRs of participating primary care repositories in five Canadian provinces and participating in the Canadian Primary Care Sentinel Surveillance Network.^36^ The Repository includes data on all patients identified as having either type 1 or type 2 diabetes using a validated algorithm.^37^

### UK — England

Data from the Royal College of General Practitioners Research and Surveillance Centre (RCGP RSC) were used, one of Europe’s oldest sentinel networks, and in 2019 is in its 53rd year.^38^ Its primary role is monitoring flu, other infections, and measuring vaccine effectiveness.^39^ It has also been extensively involved in diabetes research^40^ and captures cardiovascular outcomes.^41^

### UK — Scotland

The Scottish Care Information — Diabetes Collaboration database was used, which includes nearly all patients in Scotland with diabetes.^42^ It includes data from primary care (visits, laboratory tests), hospital diabetes clinics, community care, and retinopathy screening.

### Study population

The population of interest was persons, aged ≥40 years, living with type 2 diabetes defined as receiving at least one glucose-lowering medication for each year of interest. Receipt of one of these medications was defined as receiving a prescription in Canada and the UK, and as receiving a dispensing in Australia. Patients aged <40 years were excluded as younger persons have a greater likelihood of being diagnosed with type 1 diabetes.^43^ Also, patients receiving insulin only were excluded, as they were more likely to have type 1 diabetes rather than type 2.

### Ascertainment of glucose-lowering medication use

Patients using glucose-lowering medications from each class were identified as those with at least one drug provided for each class of medication during each year of interest. The classes of medication were: metformin, SUs, DPP4, insulin, SGLT2s, GLP1, glitazones, and other (acarbose, meglitinides).^8^^,^^9^ Medications in these classes were identified from prescription and dispensing records according to the anatomical therapeutic chemical (ATC) codes provided in Supplementary Table S1. Medications prescribed as combinations in a single pill were counted as if each separate class had been provided. Insulins were excluded from combinations of medications as this class is available without a prescription in several Canadian provinces.^43^

### Covariates

Available variables reported from all nations included age ranges and sex as of 31 December 2017. Relevant laboratory values are reported in the present study: vital signs (blood pressure, body mass index), number of comorbidities, and number of encounters in Canada and the UK. Information on ethnicity was available for EMR data in the UK only.

### Statistical analysis

To report patterns of prescribing (dispensing in Australia) over time, the authors examined the proportion of the study population on each class of medication in each nation for each year of interest using descriptive statistics. The numerator was the number of patients with at least one medication in a drug class of interest in each year. They also reported proportions for each data source with respect to patients’ sex and age range. The 95% confidence intervals (CIs) for the difference in two proportions were generated using normal approximation and pooled variance.

To study whether the newer classes are complementing or replacing older drugs, the authors described trends in the proportions of the population taking medications as sole agents. To examine combinations of medications on a foundation of metformin, proportions of patients on only two (double therapy), three (triple therapy), or four (quadruple therapy) classes of medications of interest during each year were calculated. Statistical analyses were conducted using SAS software (version 9.4).

## RESULTS

**Figure 1.**
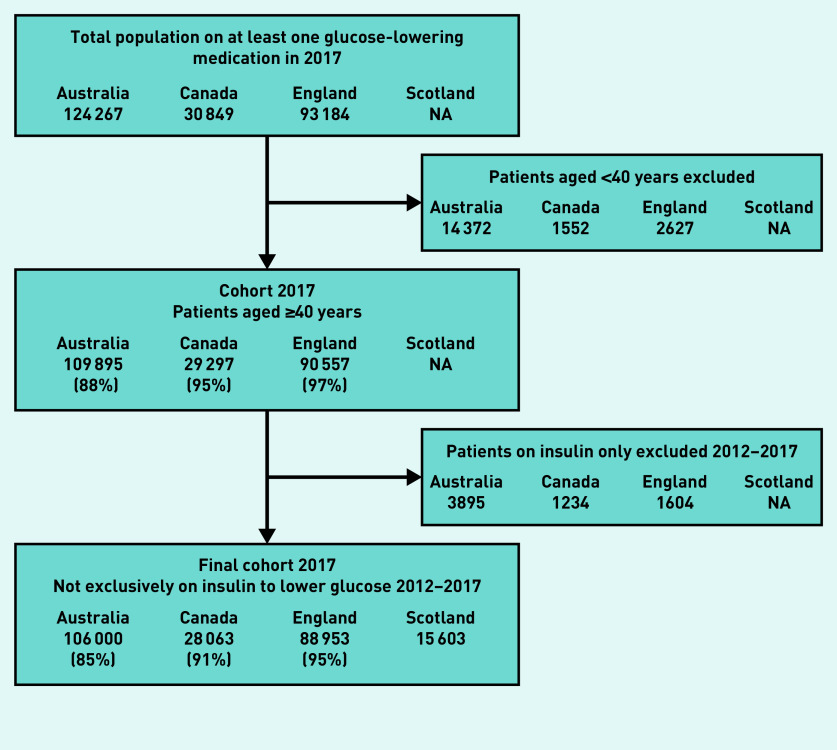
*Flow diagram for generation of cohorts in 2017. Percentages reflect: Numerator: number of patients remaining in cohort. Denominator: number of patients at cohort inception. For example, the England cohort starts at 93 184 patients. Remove 2627 patients aged <40. Remainder is 90 557. (90 557/93 184) × 100 = 97%. NA = not applicable.*

Data from 238 619 patients were included by 2017 in 2017: 106 000 patients in Australia, 28 063 in Canada, 88 953 in England, and 15 603 in Scotland. A flow diagram for cohort generation in 2017 is shown in Figure 1.

Patient characteristics are shown in Supplementary Table S2. Percentages of patients receiving each class of medication are shown on Figure 2a; percentages of patients using metformin are shown on Figure 2b as it was prescribed to a much larger proportion of patients than any other medication class. Denominators for each year and each nation, and rates of medication use, are also presented in Supplementary Table S3 (percentages add up to more than 100% as patients may be on several medication classes simultaneously).

**Figure 2a.**
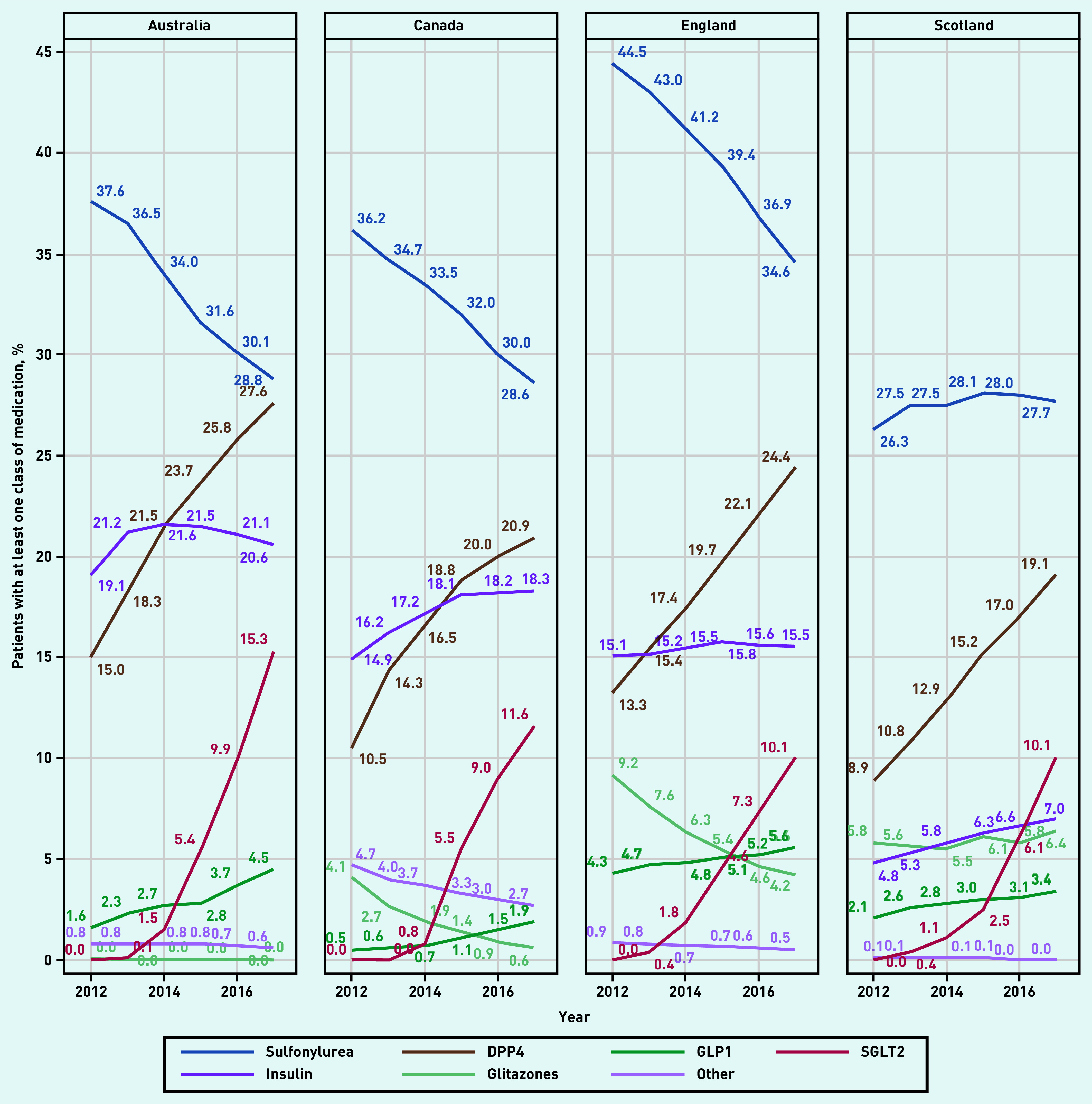
*Proportion of patients with at least one prescription/dispensing for a glucose-lowering medication in each year, according to class of medication.* *DPP4 = dipeptidyl peptidase-4 inhibitor. GLP1 = glucagon-like peptide 1 receptor agonists. SGLT2 = sodium-glucose cotransporter 2 inhibitor.*

**Figure 2b.**
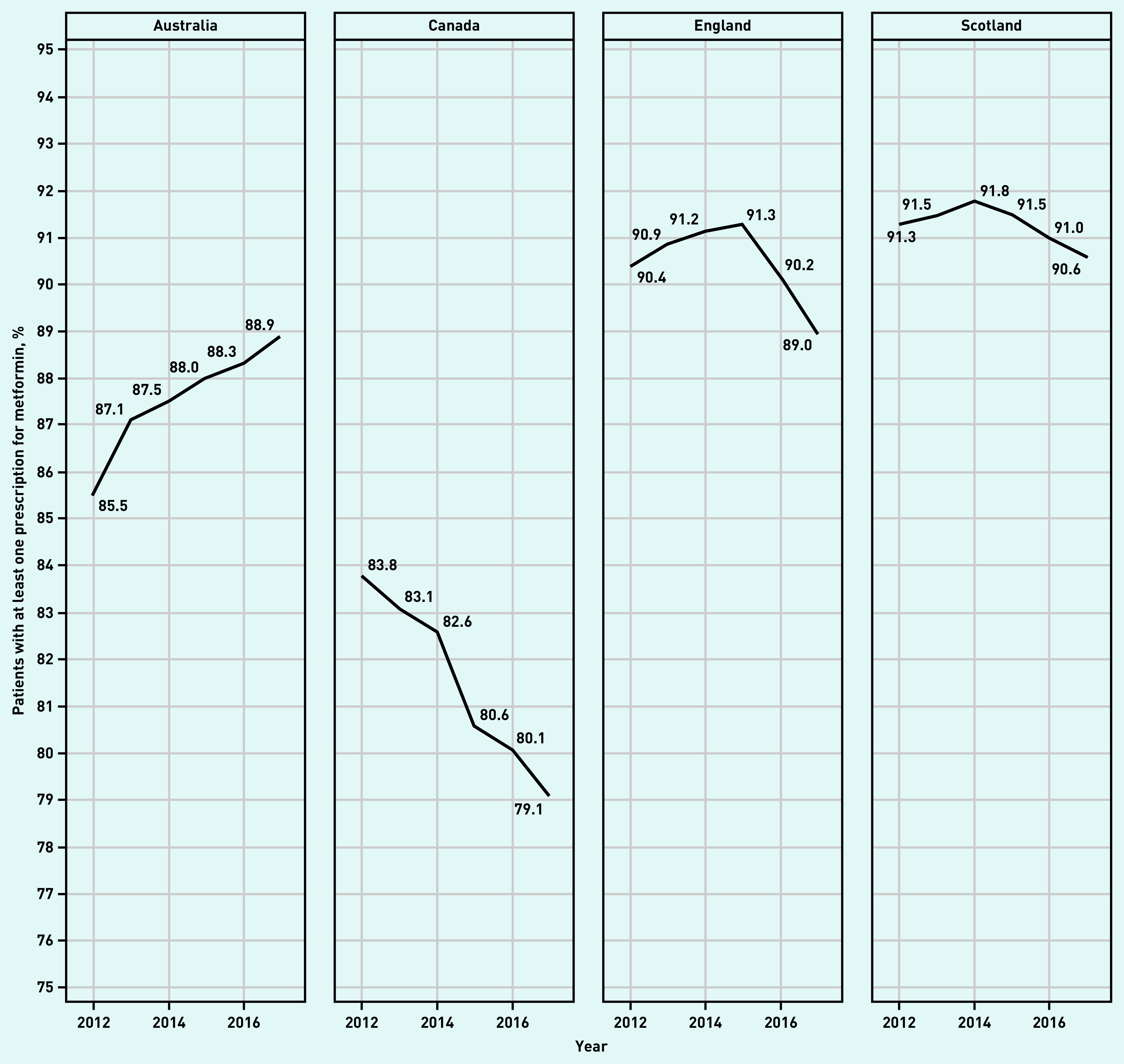
*Proportion of patients with at least one prescription/dispensing for metformin in each year.*

The overall change from 2012 to 2017 for metformin, sulfonylureas, DPP4s, SGLT2s, and GLP1s is shown on Table 1. The use of SUs decreased over the 6 years of observation in three out of four nations; metformin use decreased in Canada by 4.7%; 95% CI = 5.05 to 4.34.

**Table 1.** Change in proportion of patients on metformin, sulfonylureas, dipeptidyl peptidase-4 inhibitors (DPP4s), sodium-glucose cotransporter 2 inhibitors (SGLT2s), and glucagon-like peptide 1 receptor agonists (GLP1s) between 2012 and 2017

**Medication class**	**Nation**		**Change in proportion of patients on medication, % (95% CI)**
**Metformin**			
	Australia	3.4	(3.24 to 3.55)
	Canada	−4.7	(−5.05 to −4.34)
	England	−1.4	(−1.60 to −1.30)
	Scotland	−0.7	(−1.16 to −0.29)

**Sulfonylurea**			
	Australia	−8.8	(−9.00 to −8.59)
	Canada	−7.6	(−8.10 to −7.18)
	England	−9.9	(−10.10 to −9.63)
	Scotland	1.4	(0.77 to 2.02)

**DPP4**			
	Australia	12.6	(12.41 to 12.79)
	Canada	10.4	(10.07 to 10.73)
	England	11.1	(10.96 to 11.34)
	Scotland	10.2	(9.68 to 10.71)

**SGLT2**			
	Australia	15.3	(15.17 to 15.43)
	Canada	11.6	(11.38 to 11.82)
	England	10.1	(9.94 to 10.16)
	Scotland	10.1	(9.75 to 10.45)

**GLP1**			
	Australia	2.9	(2.81 to 2.98)
	Canada	1.4	(1.30 to 1.50)
	England	1.3	(1.17 to 1.39)
	Scotland	1.3	(1.06 to 1.54)

Although SGLT2s were rarely prescribed in 2012, by 2017, between 10.1% and 15.3% of patients were on that class. DPP4 usage ranged between 19.1% and 27.6% in 2017 (Figure 2a).

Patterns of prescriptions by patient sex are shown in Supplementary Figure S1a and S1b, and in Supplementary Table S4; changes were similar by sex. Patterns by age ranges are shown in Supplementary Figure S2a and S2b and Supplementary Table S5. The uptake of SGLT2s was highest among younger patients (aged 40–60 years).

The prevalence of sole agents is shown in Supplementary Figures S3a and S3b. About half of all patients were on sole agents for the drugs studied. Between 88.9% and 96.2% of patients on sole medications were on metformin in 2017 (percentages are not shown in tables). In Canada, the use of metformin as sole agent decreased by 5.1% while the use of either DPP4s or SGLT2s as sole agents increased by 2.8%, to 3.9% of patients on any glucose-lowering medication (Supplementary Figure S3). In Canada in 2017, 7.4% (2012: 48.2%, 2017: 43.1%, difference −5.1%, 2012: 1.1%, 2017: 3.9%, difference: 2.8%) of patients using a sole agent were on either a DPP4 or a SGLT2; the other nations adopted newer medications as sole drugs at a lower rate.

**Figure 3.**
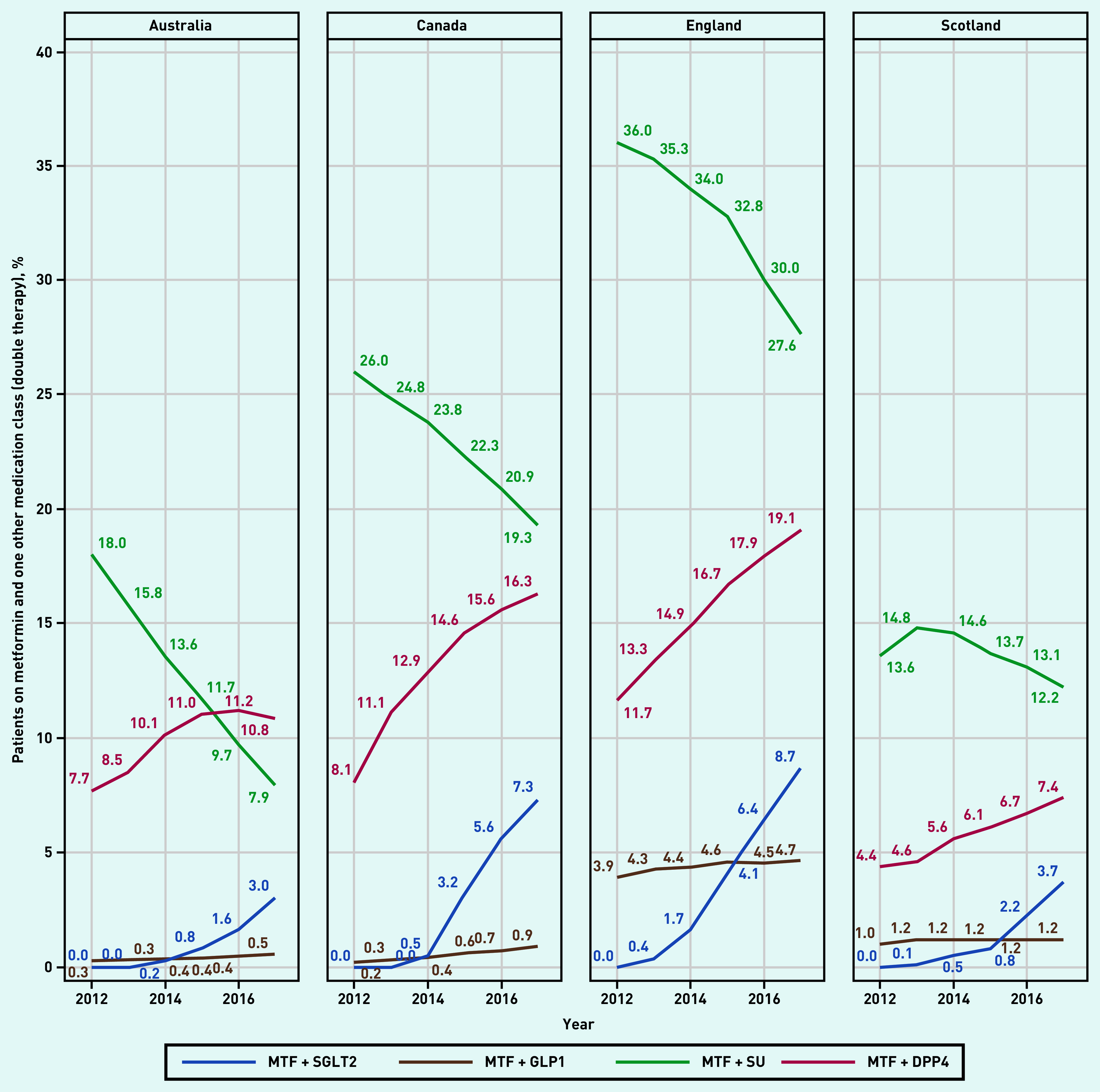
*Proportion of patients on metformin and one other medication class (double therapy). DPP4 = dipeptidyl peptidase-4 inhibitor. GLP1 = glucagon-like peptide 1 receptor agonists. MTF = metformin. SGLT2 = sodium-glucose cotransporter 2 inhibitor. SU = sulfonylureas.*

Percentages of non-insulin medications provided on a foundation of metformin (double therapy) are shown in Figure 3, and in Supplementary Table S6a. The proportion of patients on double therapy decreased by 3.8% in Australia and increased by 9.5% in Canada, 8.5% in England, and 5.5% in Scotland (Supplementary Table S6a). The proportion of patients on combinations of metformin and SU decreased in all settings, whereas combinations of metformin and DPP4s increased in all settings, ranging from 7.4% to 19.1% of patients in 2017. Combinations using SGLT2s were rare in 2012; in 2017, these ranged from 3.0% to 8.7% of patients. The increase in metformin–SGLT2s combinations between 2015 and 2017 outpaced that for metformin–DPP4s by 2.4% in Australia and Canada, 2.2% in England, and 1.6% in Scotland.

In 2017, use of either a DPP4 or a SGLT2 as double therapy on a foundation of metformin approached or exceeded the use of an SU. The percentage of patients on either new drug as combination therapy with metformin ranged from 11.1% to 27.8% (percentages can be calculated from Supplementary Table S6a). Information on triple and quadruple non-insulin therapy on a foundation of metformin (selected drug classes) is presented in Supplementary Tables S6b–c. Triple therapy with the drugs in question increased in all nations. Quadruple therapy was found in <2.5% of patients in 2017, for all nations.

## DISCUSSION

### Summary

The presented comparison across Australia, Canada, England, and Scotland demonstrates substantial and rapid changes in glucose-lowering medication use for type 2 diabetes between 2012 and 2017. Although most patients were on the recommended first-line medication, metformin, use of this drug decreased in Canada. Newer drugs were displacing SUs, with similar trends in all four nations; DPP4s were used more frequently than SGLT2s or GLP1s, despite evidence of more favourable outcomes for the latter two in preventing adverse cardiovascular outcomes.

The finding of a decrease in metformin use in Canada was surprising and has not been reported elsewhere to the authors’ knowledge; this observation should be replicated in other studies, as guidelines continue to recommend this drug as first-line treatment.^8^

### Strengths and limitations

The authors used routinely collected data from community-based primary care and included a large sample of both patients and primary care providers from multiple settings across Australia, Canada, and the UK, observed over 6 years. Therefore, this study reasonably reflects current clinical practices for individuals receiving primary care in the settings studied, all of which have universal, publicly funded health care with primary health care as foundational elements.

The EMR data were a convenience sample of primary care practices that contributed data in Canada and the UK, and may not necessarily be representative. Medications from specialists and other providers were not included in those datasets; some family physicians may not be entering prescriptions correctly into their EMRs. Diabetes guidelines currently recommend SGLT2s or GLP1s in patients at high cardiovascular risk; however, the authors were unable to segment this population owing to data limitations.

### Comparison with existing literature

Two recent studies found high rates of initial use of metformin in the UK, followed by replacement of SUs with DPP4s and SGLT2s as additional medications.^24^^,^^25^ A similar displacement of SUs as second line by the newer drugs was observed in Denmark, Finland, Norway and Sweden.^44^ Trends were similar using EMR-based prescribing data in the US^23^ and in Medicare beneficiaries; costs are rising rapidly, with the most costly non-insulin category being DPP4s.^45^ These trends were confirmed in the nations included in the presented study, with DPP4s and SGLT2s replacing SUs as combination medications.

The authors found that the uptake of SGLT2s was most pronounced in younger patients. A recent study using US claims data similarly found higher rates of adoption in younger patients with a lower risk.^46^ Current guidelines recommend these drugs for patients at greater cardiovascular risk.^8^^,^^9^ Absolute risk reduction with any therapeutic intervention depends on baseline risk,^47^ and cardiovascular risk associated with diabetes increases with age.^48^ Expanding the use of newer medications more rapidly in younger populations presumed to be at lower risk may dilute the overall effect. Longer life expectancy for younger persons entails greater medication costs over time; this may be balanced by larger decreases in cardiovascular outcomes owing to longer use.

### Implications for research and practice

Rates of adverse cardiovascular outcomes were found to be higher after initiation of SUs or insulin as second-line medications, compared with DPP4s, SGLT2s, or GLP1s.^17^^,^^18^^,^^49^ The switch to newer agents, observed in this study and others, seems clinically sensible. SGLT2s and GLP1s have been associated with better cardiovascular outcomes, lower mortality, and more favourable effects on patient weight than DPP4s.^4^ The present study found that DPP4s are still used more frequently than SGLT2s or GLP1s; clinicians may be reconsidering this, as combinations of metformin–SGLT2s increased faster than metformin–DPP4 combinations from 2015 to 2017.

There are additional costs of shifting to newer drugs, incurred by health systems when coverage is provided, and by individual patients lacking drug coverage.^50^^,^^51^ A recent study found that covering a set of essential medications at no charge to patients resulted in better adherence, with patients reporting improvements in their ability to make ends meet (buy food, pay rent).^52^ Tradeoffs in costs and outcomes, for patients and health systems, of the rapid uptake of these medications in broad populations of persons with diabetes should be studied further.^53^

It would be helpful to continue to monitor the extent to which the opportunity to switch to medications with evidence of better outcomes is seized in primary care settings. Diabetes associations that develop guidelines have an important role in setting targets for adoption and could support and fund regular reports to monitor progress in primary care. Barriers to change in the management of diabetes in primary care are well known, and include context, resources, skills, knowledge, and emotions.^54^ Strategies can involve individual, collective, and structural changes;^55^ these include prioritising the change, leadership support, presence of champions, and audit and feedback.^56^^–^^58^
